# Supplementary material for: Are carbon emissions trading and green financial instruments synergistic? -Comprehensive quantitative research based on content analysis
Source: PLoS One. 2024 Mar 7;19(3):e0298601. doi: 10.1371/journal.pone.0298601 (PMC10919601; doi:10.1371/journal.pone.0298601)
Supplement: S1 Data — (ZIP) [file pone.0298601.s001.zip › Data/地级市Z_F工作报告.zip!/┴╔─■/╕╖╨┬╩╨/└┤╫╘╣1⁄2╓┌║┼í╛┬φ┐╦╩2╛▌═°í┐_2003.docx]

各位代表：

　　现在，我代表市人民政府，向大会报告工作，请予审议，并请市政协各位委员和其他列席同志提出意见。

　　一、2003年全市人民团结奋斗，国民经济和社会发展取得显著成就

　　2003年，是阜新发展史上极不平凡的一年，是我们战胜非典侵袭和历史上特大春旱等诸多困难，经受住各种考验和挑战的一年，是我们紧紧抓住发展这个第一要务，全力推进阜新转型与复兴，实现经济跨越发展的一年。一年来，在市委的正确领导下，在市人大、市政协的监督支持下，市政府团结和带领全市各族人民，高举邓小平理论和“三个代表”重要思想伟大旗帜，抢抓机遇，顽强拼搏，团结奋斗，经济转型继续向纵深推进，国民经济保持高速协调发展，各项社会事业取得明显成就。

　　国内生产总值越过百亿元大关，成为阜新经济发展史上新的里程碑。预计全市国内生产总值达到104.2亿元，比上年增长20.5%，其中，第一产业增加值22.3亿元，增长45.2%；第二产业增加值40.7亿元，增长18.6%；第三产业增加值41.2亿元，增长12.1%。全社会固定资产投资50亿元，增长21.4%。地方财政一般预算收入5.13亿元，增长15%。城镇居民人均可支配收入5050元，农民人均纯收入2050元，分别增长8.1%和28.8%。经过“十五”前三年的不懈努力，阜新经济开始进入一个新的快速发展时期。

　　过去的一年，着力抓了以下12个方面工作。

　　1、突出抓好重点项目建设和龙头企业发展，经济转型向纵深推进。23个经济转型重点项目进展顺利，原种猪场、细河城市段治理、垃圾处理厂、农网二期等项目竣工，海州立井、清河门矿井改扩建和污水处理厂开工建设；引白水源一期工程项目顺利通过中咨公司的评估，彰武火电项目通过省专家组初可研评审，阜矿集团煤矸石热电项目上报国家发改委。阜新双汇、阜新大江和比牛哥乳业等一批代表转型形象的大型龙头企业建成投产，经济转型又一重大龙头企业项目内蒙古草原兴发落户阜新；同时，新引进上海星辉、美中鹅业、亚洲红冰葡萄酒、沈阳禾丰等一批知名龙头企业。转型农业园区达到20个，专业小区达到55个，创造就业岗位8000多个。阜新国家农业科技园区主要功能区基本达到“七通一平”，新建3个科技研发中心。采煤沉陷区治理新开工面积35.4万平方米，5360户沉陷区居民喜迁新居。

　　2、加大对内对外开放力度，利用外资和出口创汇首次越过2000万美元大关。全市招商引资到位26亿元，增长25.6%，其中直接利用外资2230万美元，增长31.2%。重大项目招商引资取得历史性突破，沈阳医联药业公司生物制药项目一期工程开工建设，香港投资的信息产业大厦破土动工，台商投资的一次性自毁针头注射器项目签订合同并立项报批；林纸板一体化和双面不锈钢复合板等投资10亿元以上的项目签订协议。继续扩大外引内联，积极开展赴韩国、香港、青岛、厦门、温州等境内外经贸洽谈活动，取得显著效果。对外影响进一步扩大，去年国内外客商来阜考察、洽谈项目的团组比上年增加一倍以上。按照国际市场的需求，加强出口商品基地建设，出口商品结构进一步优化。出口创汇2300万美元，增长38.2%。细河区出口创汇总额居全市两县五区之首。阜新经济技术开发区招商引资步伐加快，中德卡巴特公司搅拌站等7个投资3000万元以上项目入区。

　　3、大力推进农业和农村经济结构调整，农业生产全面丰收。战胜历史上特大春旱，粮食产量110万吨，增长18.3%；油料、杂粮、蔬菜、花卉、薯类、水果等种植面积大幅度增加。为双汇、大江和比牛哥等龙头企业配套的畜牧业基地建设取得阶段性成果。全市猪、牛、羊、禽出栏量分别增长24.5%、22.7%、47.1%和34.3%，具有国内一流水平的曾祖代猪场基础工程完工。植树造林创历史最好水平，全年造林80.4万亩，其中退耕还林65万亩。新植果树4.2万亩，人工种草10.5万亩。6大示范工程建设全面展开。建设绿色食品基地30万亩；新发展蔬菜保护地1.35万亩；新建“四位一体”生态模式户1.13万户；林下经济开发面积45万亩。新建一批农业现代化水利示范工程，新增节水灌溉面积3.1万亩，完成水土流失治理面积35.6万亩。机械化保护性耕作面积达12.3万亩，居全省首位。人工增雨8.4亿立方米。

　　4、进一步推进国企改革和工业结构调整，工业经济运行速度和效益明显提高。规模以上工业总产值完成82亿元，增长20%，工业增加值21.9亿元，增长15.9%；处理并轨费用后，实现利润2100万元，按可比口径比上年增盈6300万元。新邱区实现工业总产值居五区之首。全市31户国有企业实现转制。阜橡集团等10户企业与域外大企业、大集团实现合资合作。“两毛一纺”完成破产终结法律程序的各项准备工作，阜矿集团4个破产项目列入国家计划，地方4个破产项目列入国家建议计划。恒瑞公司500吨浮法玻璃在线银镜生产线、辽宁大金集团建筑钢结构改造等一批项目竣工投产。阜矿集团全面提升井工，稳定煤炭生产，原煤产量1215万吨，创近24年最高水平。阜新发电厂发电量40亿度，创近27年最好水平，三期改造项目进入开工前准备阶段。全面推进新型工业园区建设，玻璃、电子工业园区开始启动，橡胶工业园区项目开始进区。太平高科技园区入驻企业12户。

　　5、进一步调整结构，扩大规模，民营经济发展实现新跨越。民营经济增加值增长28%，占全市经济总量42%；实缴税金2.9亿元，增长40%；从业人员新增9200人。民营企业发展规模不断壮大，销售收入超千万元的民营企业达到45户，东祥植物油公司、春城集团销售收入超亿元，石油工具厂等10户企业参与国企转制。乡镇企业增加值、出口交货值、实缴税金分别增长50.6%、160.6%和38.5%。

　　6、加快发展第三产业，加强市场体系建设，新兴产业不断发展壮大。社会消费品零售总额实现49.5亿元，增长10.8%。阜新国泰大厦正式与大商集团合作，肯德基项目成功引进。商业系统工业对全市税收增长作出突出贡献。阜新商贸城基建工程全部竣工，阜新建筑装饰材料市场主体工程完工，辽西批发大市场绿色农贸市场投入使用。大清沟、瑞应寺风景区晋升为AA级景区。阜蒙县争创旅游强县工作通过省验收。全市住宅开工面积97万平方米。财政筹资能力进一步增强，巩固基本财源、培植新财源工作取得成效。阜蒙县地方财政一般预算收入突破亿元。国、地税工作力度加大，税收征管工作成效明显。审计工作质量不断提高，在为经济发展服务中发挥了积极作用。全市金融机构加大对经济转型项目的信贷支持力度，新增贷款16.7亿元，增长10.5%；为地方企业核销贷款本息2.17亿元。

　　7、进一步加大经营城市和基础设施建设力度，城市功能进一步完善。全市土地收益1.61亿元，其中市本级土地出让金9795万元，增长18%。细河中心游园、月亮广场工程竣工。煤城路全线通车，极大改善了城市南部地区的交通条件。实施了矿工大街、人民大街、新华路等3条城市主干路改造工程。对城市南部地区实施改造40万平方米。完成村通油路214公里，51个行政村实现通油路。长途客运站新建工程主体完工。完成了市区北出口、西北出口和西出口绿化工程，城市绿化覆盖率提高2.1个百分点，人均公共绿地面积增加1平方米。我市在省“绿叶杯”评选中获优胜单位，海州区实现城区组“十三连冠”。城市供热分户改造143.5万平方米。改造小街小巷20条，解决了市北新村等2400多户居民供热难及他拉庙等3000余户居民吃水难问题。建筑业增加值增长14.5%。小城镇示范镇建设取得进展。组织街路综合整治三大战役，取缔马路市场14处，拆除违章建筑2072处。对全市26条街路实行“门前三包”，城市管理水平有新的提高。城市空气质量全年60%的天数达到二级标准。彰武县被批准为第八批全国生态示范区建设试点县。

　　8、切实关心人民生活，社保试点任务全面完成，就业和再就业工作取得新进展。全市国有企业符合条件的职工全部实现并轨。基本养老保险参保人数增加1.2万人；基本医疗保险参保人数增加3.1万人。农村养老保险费收缴突破千万元大关；全年征缴基本养老保险费1.6亿元，失业保险费2640万元，超额完成省政府下达的征缴计划。全年发放最低生活保障金1.1亿元。实现就业和再就业4.2万人，其中安置“4050”下岗职工6500人。工会、共青团、妇联在再就业工作中发挥了重要作用。大力开展“社区建设年”活动，213个社区办公室和155个社区“一站三室”达到省定标准，适当提高了社区办公经费及干部补贴标准。解决了23万农村贫困人口的温饱问题。筹集款物8593.6万元，对9.1万户、29.9万城乡特困人口进行了救助；政府系统县级以上领导干部对409户贫困户、374名贫困生进行了包扶，帮助374名特困下岗职工实现再就业。

　　9、先行试点，稳步推进，农村税费改革进展顺利。全市核定计税常年产量旱田为261公斤，水田为379 公斤。省政府为进一步减轻粮食主产区农民负担，实行政策性减免措施，使税改后农民人均减负52.7元，减负率48.8%。全市836个行政村减少到624个；教育布局调整、乡镇事业单位改革、化解村级不良债务等配套改革正在进行中。

　　10、加快发展科技教育，为经济发展提供了智力支撑。与国内著名高等院校、科研院所的合作深入开展，一批科技成果得到应用。引进推广农业新品种65个、新技术15项。科技部“阜新现代农业科技专项”13个项目进展顺利。中国科协将阜新确定为全国依靠科技推进经济转型与振兴试点市，组织开展了“全国百名科技大王进阜新”活动。国家级农业综合开发科技推广项目效益显著。专利申请量197件，增长27.9%。全市高新技术产业产值实现10.2亿元，增长27.5%。辽工大发挥智力优势，积极支持和参与阜新经济转型。阜新高专通过资产置换，新建综合楼、学生公寓及设施3.5万平方米。成立了市高专农业职业技术学院。高中当年招生首次突破万人大关。改造农村危险校舍3.5万平方米，创建办学设施标准化乡镇6个。农村初中辍学率控制在省考核指标3.6%以内。通过实施“一村一名大学生”计划，累计为农村培养各类人才517人。

　　11、加大精神文明建设力度，各项社会事业有新的发展。通过兴起学习贯彻“三个代表”重要思想新高潮活动，为推动经济转型提供了有力的思想保证。以“建设开放阜新，争做文明市民”为主题的精神文明创建活动蓬勃开展。阜、彰两县文化中心大厦主体完工，海州区文化中心交付使用。农村有线电视“村村通”入户2 万户。成功举办了首届全国篮球城市文化交流大会。阜新篮球学校综合训练馆落成。清河门区乌龙坝镇被评为全国亿万农民健身活动先进单位。市中心医院综合病房楼投入使用。市疾病预防控制中心综合测试楼竣工。以11所重点乡镇卫生院为骨架的农村医疗网络进一步完善。社区卫生服务覆盖率达到90%。全面开展创建计划生育优质服务县区活动，人口自然增长率控制在1.63‰以内。妇女、儿童、老龄、残疾人事业和物价、档案、地方志、地震预测预报等工作取得新成绩。

12、加强民主法制建设和廉政建设，社会大局保持稳定。认真办理人大代表建议和政协提案，办结率100%，满意率90%以上。坚持依法行政，加强了行政执法部门自身建设。“四五”普法教育扎实推进。严厉打击各种刑事犯罪活动，组织开展了“严打百日会战”等一系列集中打击行动，严密防控“法轮功”等邪教组织的破坏活动，及时、妥善处置群体性事件和突发事件，创造了稳定的社会环境。在市场准入、文化市场、建筑市场、交通运输、税收等领域开展了专项整治，维护了全市经济秩序。以处理疑难信访案件为重点，落实领导包案责任制，省交办案件和市自排案件结案率分别达100%和98%。认真落实廉政责任制，加大查办违法违纪案件的力度，纠正部门和行业不正之风，廉政建设得到进一步加强。以推进政务公开、村务公开和厂务公开为主要措施，民主管理工作取得新进展。统计工作在为经济转型服务中不断强化。国防教育、民族、宗教、外事、侨务、对台、国家安全、人防、民防等工作进一步加强。

　　阜新电业局、烟草公司、石油公司上缴税金贡献突出；质量技术监督、药品监督、工商、电信、邮政、出入境检验检疫等中省直单位和部门，从各自职能出发，为促进地方经济发展发挥了重要作用。驻阜部队、武警官兵为加快阜新转型复兴作出了积极贡献。

　　过去的一年，我们经受了一场防治非典的严峻考验。在市委的正确领导下，我们坚持一手抓转型发展不动摇，一手抓防治非典不放松，全市上下众志成城，和衷共济。经过三个多月的艰苦奋战，成功地阻住了非典的袭击，实现了非典病例“零输入”目标，取得了抗击非典的阶段性重大胜利。这场没有硝烟的战争，更好地检验了我们这支队伍应对复杂局面的能力，充分显示出全市人民团结一心、战胜各种艰难险阻的强大合力。

　　过去的一年，阜新工作得到了新一届党中央领导集体的亲切关怀。胡锦涛、温家宝、曾庆红、黄菊、王兆国等中央领导同志对阜新经济转型给予了特殊关注。国家各部委、省委、省政府及省直各部门继续对阜新经济转型给予了大力支持。我们要竭尽全力，不负厚望，加快推进阜新转型复兴的伟大事业。

　　各位代表，过去一年所取得的成绩，是全市人民迎难而上、团结拼搏、开拓进取、共同奋斗的结果。在此，我代表市人民政府，向全市各族人民，向所有参与、关心和支持阜新转型复兴的同志们和朋友们，致以崇高的敬意和衷心的感谢！

　　在肯定成绩的同时，我们也清醒地看到，我市经济和社会发展中仍然存在着不少困难和问题。主要是：思想观念还不能完全适应社会主义市场经济体制的要求；经济发展结构性矛盾仍然突出；项目前期工作意识不强，项目包装水平不高；农业产业化龙头企业规模还比较小，产业化基地建设滞后；国有企业转制任务还很重，部分行业亏损面居高不下；对外开放领域不宽，招商引资还需进一步加大力度；就业工作压力较大，社会保障资金存在缺口，部分群众生活仍很困难；人才不足问题突出，劳动者素质亟待提高；软环境建设有待进一步加强，政府机关中还不同程度地存在服务质量和办事效率不高等问题。这些问题，要在今后工作中采取有力措施加以解决。

　　二、围绕阜新经济转型和老工业基地振兴，全面做好今年各项工作

　　随着国家振兴东北老工业基地战略的启动实施，阜新正面临三大历史性机遇：经济转型试点市的机遇。中央下发的11号文件明确指出，“继续做好辽宁省阜新市经济转型试点工作，总结经验，加以推广”，这是党中央、国务院对阜新经济转型的有力支持，是对阜新人民的殷切期望和庄严重托，必将为我们进一步推进经济转型试点工作创造有利条件。老工业基地振兴的机遇。阜新既是资源型城市，又是老工业基地，是整个老工业基地调整改造的重要组成部分。中央11号文件强调，要把资源型城市经济转型作为老工业基地调整改造的重点和难点，研究制定支持资源型城市经济转型的政策措施，这将在一定程度上使我市在全省和东北老工业基地振兴中处于相对重要的位置。加快辽西北发展的机遇。省里陆续出台的一系列支持辽西北地区加快发展的优惠政策，为我们加快发展县区经济创造了非常难得的契机。

　　按照市委九届五次全会精神，阜新要紧紧抓住历史机遇，以推进转型、优化结构为主线，以做大总量、提高效益和综合竞争力为重点，以观念更新和体制、机制、科技创新为先导，以扩大开放、深化改革为动力，坚持市场取向与政府引导结合、自力更生与争取支持互动、立足现有基础与发挥比较优势并举，构筑绿色农产品深加工业、煤炭电力工业、优势产业和县区经济“四大经济板块”，全面提升经济活力和可持续发展能力。经过“十五”和“十一五”的努力，从2000年到2010年，实现全市GDP总量十年翻两番，其中，2005年GDP实现150亿元，2007年实现200亿元，到2010年达到300亿元，人均15000元，超过当时全国平均水平，经济转型和老工业基地改造任务基本完成，走出一条转型复兴的新路子，为加快实现全面建设小康社会的目标奠定坚实的基础。

　　2004年是全面推进阜新转型复兴的关键一年，政府工作的指导思想是：以邓小平理论和“三个代表”重要思想为指导，全面贯彻党的十六大和十六届三中全会精神，紧紧围绕阜新转型复兴，认真落实市委九届五次、六次全会的要求，牢固树立全面、协调、可持续发展观，按照抢抓机遇、推进转型，扩大开放、深化改革，把握全局、解决矛盾的方针，以项目工作为核心，提升农业产业化水平，加速企业技术改造，加快发展第三产业，大力发展民营经济，推进“四大经济板块”建设，不断提高综合实力和整体竞争力；加强社会主义政治文明和精神文明建设，做好就业、社会保障和扶贫帮困工作，保持社会稳定，促进国民经济和社会事业持续快速协调健康发展。主要预期指标是：国内生产总值127.2亿元，增长20%；地方财政一般预算收入5.9亿元，增长15%；全社会固定资产投资61亿元，增长22%；社会消费品零售总额55亿元，增长11.1%；直接利用外资2400万美元（新口径），出口创汇2650万美元；城镇居民人均可支配收入5600元，增长10.9%；农民人均纯收入2400元，增长17.1%；人口自然增长率控制在2.3‰以内。

　　为实现上述目标，要重点抓好以下12个方面的工作。

　　（一）紧紧抓住“三大历史机遇”，打一场转型振兴项目攻坚战

　　项目是阜新转型复兴的载体、核心和重要标志。要抓住“三大历史机遇”，把项目工作作为经济工作的着力点，打一场转型振兴项目攻坚战，在全社会形成人人关注项目、关心项目、重视项目、齐心协力抓项目的浓厚氛围。

　　第一，围绕六个重点抓项目。一是围绕资源优势抓项目。突出农业资源优势，推进以绿色农产品深加工为重点的龙头企业建设，筛选、引进、争取、建设一批绿色农产品深加工项目。突出煤电资源优势，抓住国家加强电力、煤炭建设的有利时机，实施一批煤炭、电力工业项目，吸引一批用电量较高的项目和企业。发挥我市劳动力资源优势，实施一批劳动密集型产业项目，扩大就业。二是围绕比较优势产业抓项目。以汽车动力转向泵、超薄超厚玻璃、薄膜电容器、高强度橡胶运输带、聚四氟乙烯树脂、棉纺织品等在国内具有技术、设备、市场领先优势的产品为主导，包装、引进、实施一批关联度较高的产业项目。三是围绕政策导向抓项目。充分利用经济转型试点市、东北老工业基地振兴和加快辽西北发展的有关政策，编制和争取一批符合国家产业政策，能够发挥阜新比较优势的项目。四是围绕存量资产抓项目。对企业存量资产状况进行排查，摸清底数，把企业存量资产精心包装成项目，以存量引增量，以资产换资金。五是围绕国际国内市场需求抓项目。广泛收集项目信息，选择一批适应阜新转型振兴的项目，加大对外招商力度。六是围绕社会事业发展抓项目。拓宽思路，科学规划，着力抓好一批城乡基础设施建设和教育、文化、卫生、广电、体育等基础设施建设项目。

　　第二，强化六个方面工作。一是竣工项目抓验收。重点做好国家和省支持项目竣工后的验收工作，并向国家和省提出正式报告。二是建成项目抓投产。加强项目建设的动态管理，确保已竣工的项目尽快转入投产、达产、扩产。三是在建项目抓进度。明确具体时限，确保按期完工。四是新上项目抓开工。实行时间、任务、责任“三位一体”的责任制，确保尽快开工建设，早日竣工投产。五是洽谈项目抓签约。定人、定时、定责，集中力量争取签约，确保项目与资金尽快到位。六是储备项目抓可研审批。明确专人负责，积极争取国家、省有关部门的支持，确保项目申报、立项和可研审批的顺利推进。

　　第三，突出抓好六个关键环节。一是精心做好项目前期工作，加大项目前期工作投入。二是聘请省内有权威的咨询机构和专家对重点项目进行可研论证，提高项目论证的权威性。三是按照国家和省产业规划上项目，搞好与产业规划的衔接。四是充分调动项目单位的积极性，发挥金融部门在项目工作中的作用。五是落实项目的建设要素，确保项目的成功率。六是建立一套高效的项目工作体系和严格的考核评价机制及奖惩机制，明确责任，整合力量，分级负责。

　　（二）加快调整优化工业结构，推进阜新经济转型与振兴

　　围绕培育接续替代产业、优势行业和骨干企业，加快企业技术改造和结构调整步伐，推进项目建设。

　　第一，依托资源优势，大力推进绿色农产品深加工龙头企业项目建设。一是重点抓好一批竣工企业的投产达效，不断扩大规模。阜新大江形成年加工肉鸡1200万只的生产能力；比牛哥新增年加工鲜奶2万吨的生产能力；丰侨兔业、四通草业、田园实业、上海星辉、上海交大花卉、台湾福龙花卉、五彩杂粮、香香食品等龙头企业，要在达产达效的基础上，扩大发展规模。二是加快推进一批开工项目的竣工投产。阜新双汇百万头生猪屠宰线、年产2万吨低温火腿肠生产线分别在6月和8月竣工投产。辽宁沙棘药业系列产品深加工项目、宝岩芦荟深加工项目、天诚食品花生仁加工项目、钜成花卉项目、振隆公司瓜籽仁加工等一批项目年内竣工投产。三是抓紧一批项目的开工建设。草原兴发一期工程和美中鹅业一期工程等项目开工建设；力争开工建设阜新双汇年产3万吨低温火腿肠生产线，形成年加工肉制品9.5万吨的生产能力。四是加大工作力度，充分利用转型试点市效应，继续强力推进龙头企业的引进和培育，年内再引进一批国内外知名龙头企业。

　　第二，依托传统优势，搞好国家能源基地和全省装备制造业配套基地建设。一是建设一批煤炭、电力工业项目。海州立井、清河门矿井改扩建项目年底前竣工投产，争取已批复的五龙矿井改扩建项目及早开工建设。开工建设投资26.9亿元的阜新发电厂三期改造工程，争取投资25.5亿元的54万千瓦阜矿集团煤矸石热电项目被国家批复立项并开工建设，力争彰武火电、开发区热电和新邱区热电等项目立项。利用矿区资源优势，实施煤层气三期、矿井水综合利用等一批非煤产业项目。二是建设一批全省装备制造业配套基地项目。阜新德尔机械制造有限公司在年产20万台汽车动力转向泵的基础上，今年要达到30万台规模，争取年产40万台具有国内领先水平的可变排量汽车转向泵项目立项；液压件厂与韩国合资生产30万台汽车动力转向泵项目开工建设；太克液压公司生产各类汽车动力转向泵形成5万台能力；金昊公司形成为机械制造业加工中心配套各类单螺杆空压机5000台的生产能力；利用大吨位压铸机的优势，提高为各类整机、汽车配套压铸件和机加工件能力。

　　第三，依托产业优势，做大做强具有比较优势的产业。一是依托玻璃工业园区，壮大玻璃深加工业。恒瑞公司500吨浮法玻璃生产线达产达效，同时，建设年产15万套汽车风挡玻璃生产线等三个玻璃深加工项目。争取再上一条日熔化500吨浮法玻璃生产线。二是依托橡胶工业园区，发展橡胶制品业。与江阴法尔胜集团合作投资的橡胶运输带项目竣工投产；与日本横滨橡胶和三井物产株式会社合资生产700万平方米橡胶运输带项目取得实质性进展，同时，开工建设阜新连新塑胶发展有限公司年产1800吨高密度聚乙烯管项目。三是依托氟化工园区，振兴含氟精细化工业。开工建设金凯化工公司年产700吨氟化物系列产品项目。抓好凯莱英医药化学技术有限公司总投资1000万美元的医药中间体项目，确保一期工程投产达产，开工建设二期工程。四是依托电子工业园区，做强新型电子元器件制造业。东新电容器有限公司年产1.5亿只片式电容器项目达产达效，开工建设阜新宏成有限公司年产20万件管道远程自动控制器项目。

　　第四，依托产品优势，调整改造新型建材、制药、纺织、毛革制品业。辽宁大金集团年产40万平方米建筑钢结构改造、阜新超懿集团年产5000锭精编织配套工程等项目达产达效，续建仙鹤中药制药有限公司年产益心复脉2250万袋和脑血康2300万片等心脑血管系列药物GMP改造、阜蒙县蒙药厂GMP扩建改造、阜新洛斯顿化联化工厂年产100吨医药农药中间体、珂曼服饰有限公司新增服饰生产能力36万套的珂曼工业园等项目。

　　（三）以农民增收为核心，推进农业结构调整，加快发展现代农业

　　高度重视和认真解决“三农”问题，按照市场化、标准化和产业化方向，围绕畜牧业、特色种植业、设施农业和林果业四个优势产业，按绿色食品标准组织生产，大力推进农业结构调整，促进农民增收。

　　第一，实施“畜牧富民工程”。把发展畜牧业作为农业结构调整的突破口，作为带动农民增收的龙头产业来抓。重点抓好与阜新双汇、大江、草原兴发、比牛哥等12个重点龙头企业配套的畜牧业生产基地建设，新建一批标准化饲养小区、专业村和规模饲养场。新增出栏瘦肉型猪30万头、奶牛9000头、肉鸡1000万只、肥羔羊60万只、白鹅600万只、兔100万只。同时，积极抓好牧草、杂粮、花生、蔬菜、食用菌、花卉基地建设，人工种草新增10万亩，种植杂粮60万亩、花生100万亩、特菜1.2万亩、食用菌450万柱、花卉1500亩。

　　第二，实施“种植业结构优化工程”。重点发展饲料作物，加快发展高油大豆、专用玉米、药材等特色种植业，发展专用玉米50万亩、高油大豆30万亩。继续发展蔬菜保护地生产，新建“四位一体”生态模式户1万户，无公害和绿色食品面积达到100万亩，绿色食品认证10个。

　　第三，搞好生态建设工程。全年植树73万亩，其中，退耕还林60万亩，“三北”四期造林13万亩，突出抓好“三杏”、葡萄、南果梨、沙棘等经济林建设，高标准完成环城绿化带建设任务。新发展果树4万亩。

　　第四，搞好农业基础工程建设。新建库塘井窑等集水工程1500项，增加节水灌溉面积3万亩，新建农村基本饮水井541眼，完成水土流失治理35万亩。引进推广先进适用农机技术，提高农业机械化水平。加强农村经纪人队伍建设，提高农民进入市场的组织程度。

　　第五，实施“四线百园”工程。以畜禽饲养、设施农业、林果、草业为重点，在101线、阜沈、阜锦公路和阜锦高速公路沿线4个产业带建设100个各具特色的专业及综合园区。

　　第六，推进五大示范工程。建设农业生产标准化示范点10个、有机食品示范点6个、林下经济示范点50个、“四位一体”生态模式户示范点15个、节水示范点4个。强化绿色食品检测体系、技术服务推广体系、无规定疫病示范区缓冲区建设体系建设。

　　第七，抓好五大繁育中心建设。继续推进瘦肉型猪繁育、肉羊繁育、种兔繁育、种驴繁育和种苗繁育中心建设。建成市曾祖代猪场、奶牛良种繁育场、良种鹅繁育场等重点项目，完善肉鸡、肉羊和肉驴繁育体系建设，重点引进、培育和推广蔬菜、粮油、林果新品种。

　　（四）以市场体系建设、旅游业和社区服务业为重点，加快发展第三产业

　　按照拓宽领域、扩大规模、提升档次的原则，突出抓好市场体系建设、旅游业和社区服务业三大重点，力争第三产业发展有一个较大突破。一是积极推进大型商贸流通企业走联合之路，壮大实力，提高效益。继续推进阜新国泰大厦与大商集团合作，完成大商集团阜新新玛特购物广场改造并投入使用。二是加快商贸基础设施的改造升级，阜新商贸城、阜新建筑装饰材料市场在上半年投入使用。同时，开工建设阜新电子商城和农产品批发专业市场。三是推进流通体制改革，加快中小型商业企业退出国有序列，年内有60%小型国有商业企业实现转制。粮食系统要深化改革，充分发挥特有优势，在发展粮食深加工业等4个产业上取得明显成效。四是整合旅游资源，积极培育阜新特色旅游品牌，开发矿区特色旅游资源和宗教特色旅游资源，加快旅游业发展步伐。五是结合城市南部地区改造，稳步发展房地产业，全市住宅竣工面积[Page]70万平方米。六是加强社区服务网点建设，不断拓展社区服务业发展空间，充分发挥社区发展经济、承载就业的功能。

　　认真做好财税金融工作。一是加快财源建设，优化支出结构。各级财政要继续把巩固基本财源、加快培植新财源作为财政工作的重点，依法从严治税，加强非税收入征管，确保财政收入增长目标的实现。同时，积极争取国家和省专项资金，优化支出结构，确保公教人员标准工资、社会保障资金和事关转型发展项目的支出。二是进一步强化财政监督和审计监督，特别是要把项目资金使用情况作为监督的重点，坚决用好管好各项资金。三是各级金融部门要加大对全市重点项目的信贷支持力度，继续做好企业核销不良贷款及不良资产工作。做强做大市商业银行，改善股本结构，增强竞争能力，积极利用再贷款，支持经济发展。

　　（五）以招商引资和体制机制创新为重点，坚定不移地推进改革开放

　　以项目为核心，推进全方位对内对外开放。一是突出重点国家和地区招商，积极组织参加省政府举办的12次境内外招商活动，重点抓好对韩国、日本和香港的招商，推进对台湾、新加坡的招商，增强招商的实效性。二是扩大外引内联，依托重点产业、优势产品和各类园区，抓住国内上海、广东、温州、沈阳、大连等发达地区产业转移和战略布局的重要契机，积极主动承接和吸引一批项目。三是全力推进林纸板一体化项目、西班牙独资年产30万吨双面不锈钢复合板项目、阜矿集团与香港合资建设黑索今项目等一批重大利用外资项目的合资合作，开工建设台湾独资年产4亿支一次性自毁针头注射器项目，香港独资信息产业大厦主体工程完工。四是积极应对国家将实行新的出口退税政策和国际贸易保护主义加剧等新形势，进一步调整出口商品结构，鼓励和支持企业扩大出口，紧盯国际市场需求，建设一批新型特色出口产品基地，培育和壮大一批新的出口大户，千方百计扩大出口。五是充分利用国家的鼓励政策，支持有实力的企业走出去，建立海外生产基地。积极开拓国际劳务市场，发展对外劳务合作。六是充分发挥阜新经济技术开发区和国家农业科技园区的先导作用，加快与国际接轨进程，重点抓好一批外资项目的实施，力争在引进重大项目和利用外资上实现新的突破。

　　以十六届三中全会精神为指导，全面推进各项改革。一是继续推进国有工业企业改革。对25户国有企业进行转制，使全市国有企业全部完成转制，其中股份制企业达到60%以上。国有大中型企业力争在投资主体多元化方面有大的突破。规范转制操作程序，确保国有资产不流失、职工利益不受损、银行债务不悬空。对转制企业的卸任经营者予以妥善安置。推进阜新电缆厂等10户企业同域外大企业、大集团联合挂靠。继续做好企业不良贷款核呆工作。对王营煤矿等9户企业实施破产，争取6户企业列入国家破产计划。二是创新企业管理方式和管理体制，树立品牌意识，争创一批名牌产品，提高产品竞争力。继续推进厂务公开，充分发挥工人阶级的主人翁作用。切实抓好企业扭亏增盈，规模以上工业实现利润2600万元，增长23.8%。三是深化农村各项改革。巩固农村税费改革成果，完善各项配套改革措施。加快农村五站体制改革；推进农村投融资体制改革；继续推进农民土地承包经营权流转和林业、小型水利设施产权制度改革。

　　（六）以大力发展民营经济为主，做大做强县区经济

　　按照以民营为主，大力推进工业化、产业化、城镇化的要求，立足县区资源状况和产业发展趋势，着力打造区域特色主导产业。各县区要认真研究、用好用足用活加快辽西北发展政策和省支持县域经济、民族地区、贫困地区发展的一系列政策措施，加快增强综合实力。继续加大支持县区发展力度，制定出台相应政策，为县区创造良好的发展环境。年内，每个县区至少引进和开工建设3个固定资产投资在3000万元或300万美元以上的生产加工型项目。

　　把发展民营经济作为全市经济发展的战略重点，调动全社会力量，加快培育和壮大民营骨干企业群，扩大民营经济总量。引导民营企业围绕转型振兴，向重点行业、支柱产业方向拓展领域；鼓励民营企业向一、二产业延伸，参与国有企业重组，在发展新兴产业中挑大梁；大力培育机械、油脂、服装、电子等经济实力强、技术水平高、地区经济特色突出的优势行业和骨干企业，支持有条件的民营企业做大做强。全市新增销售收入超千万元的民营企业10户，其中超亿元的2户，民营经济占全市GDP的比重达到60%以上。推动乡镇企业向工业园区集聚、向小城镇集中，在促进农业产业化经营中发挥积极作用。全市乡镇企业增加值、实缴税金分别增长20%和15%。

　　（七）大力发展科技教育，搞好人才队伍建设

　　巩固发展与省农科院、中国农大、沈阳农大和中国农科院等科研院所及高校的科技合作，做好省农科院阜新分院建立的准备工作。引进农业新品种100个，推广农业新技术15项。科技部“阜新现代农业科技专项”在经济转型中要充分发挥效益，加强经济转型科技项目对接洽谈会签订项目的跟踪和落实，作出阶段性总结。积极推进中国科协支持的依靠科技推进经济转型与振兴试点市工作。国家农业科技园区的农业科技综合楼上半年启用，各类研发中心要突出特色，形成产业，加快研发成果转化、推广和应用。加强科技创新体系建设，做好知识产权工作，加快发展高新技术产业，全市高新技术产业产值实现12亿元，增长17.5%。

　　充分发挥辽工大、阜新高专等高校在全市转型复兴中的重要作用。支持辽工大适应新形势，调整学科结构，扩大办学规模。搞好阜新高专3+2对接本科形式的联合办学，建设农学、园艺、林学等一批骨干专业。完成大中专院校和中等职业教育布局调整，实现教育资源优化配置，为转型复兴培养更多的实用人才。巩固高中扩招成果，推进示范性高中建设。以五个城区为重点，理顺以区级政府管理为主的义务教育管理体制。实施中小学微机联网“校校通”、农村危险校舍改造等一批教育实事工程。结合税费改革，全面调整农村中小学布局。大力发展农村职业教育，重点抓好阜、彰两县职业教育中心建设。认真落实人才政策，积极营造有利于人才成长、人才聚集的环境。推进人事制度改革，发挥现有人才的作用，不拘一格地评聘各类人才。建设市人才市场，为人才开发、引进、推介、交流创造条件。继续抓好辽工大等高校对阜新定向招生和“一村一名大学生”人才培养计划的落实。加大定向为企业培养所需技术工人的力度。采取有效措施，进一步做好引智工作。

　　（八）实施“十大工程”，大力改善城乡人居环境

　　围绕进一步完善城市功能，提升城乡整体形象，重点实施“十大工程”：（1）实施小街小巷治理工程。对市区28条小街小巷进行综合整治，主要街路消灭无灯路。（2）加快城市南部地区改造工程。对平房区实施改造，抓好棚户区改造，逐步消灭危房、平房，特别是要把煤城路两侧改造与安置沉陷区居民结合起来。（3）实施道路改造工程。对红树路东段、八一路东段等街路进行改造。（4）启动站前区域综合整治工程。抓好平房区改造、站前广场改造及火车站周边环境整治，使站前中心区域成为阜新对外开放的窗口。（5）推进一批重大基础设施建设工程。启用新长途客运站，开工建设海新路七号桥，启动阿金公路跨线桥工程。新开采煤沉陷区治理建筑面积22万平方米，基本完成一期治理工程。完成25.3公里供水管网改造工程。（6）实施城市出口路整治工程。重点对国铁城市段和市区三个出口路两侧环境进行美化、绿化、亮化。（7）实施城市园林化工程。在东起东环路、西至迎宾大街、北起八一路、南至红树路城市中心区小内环范围内，进行花园式城市建设，提高绿化档次和水平，年内新增绿地80万平方米，城市绿化覆盖率达到40%，人均公共绿地面积达到6.4平方米。（8）实施城市管理创新工程。着力建立依法管理、长效治理机制，强化城市规划管理，全面加强城市街路道板、绿地、树木及环境卫生的管理，使城市管理跃上新水平。（9）实施环境污染治理工程。在严格执行“三同时”制度的同时，建立环境保护“绿色通道”，解决好大气、水、粉尘、噪声等污染问题。本着“依法、从重”的原则，加强细河上游污染源治理。抓紧搞好环保功能分区规划。（10）推进小城镇建设工程。抓好6个重点示范镇建设，增强乡镇综合实力。完成村通油路423.7公里任务，解决79个行政村通油路问题。

　　（九）构筑就业、社保、扶贫帮困“三位一体”的工作体系，认真解决关系人民群众切身利益的问题

　　继续高举创造就业机会的旗帜，进一步采取有力措施，拓宽就业和再就业渠道。一是继续实施“一把手”工程，进一步落实目标责任制，确保全年实现就业3万人。二是进一步完善就业服务体系建设，认真落实促进就业各项扶持政策，建设市劳动力市场，为扩大就业创造条件。三是通过大力引进龙头企业、放手发展民营经济、强化转型基地建设、发展就业容量大的劳动密集型企业、创业实体和社区服务业，努力增加就业岗位。扩大劳务输出，全年输出城乡劳动力8万人，其中城镇下岗失业人员8000人。四是高度重视再就业培训工作，提高下岗失业人员就业技能和创业能力，全年培训2万人。五是统筹城乡就业，保障进城务工农民的合法权益，切实解决用工单位拖欠农民工工资问题。

　　巩固社保试点成果，加强“三条保障线”的衔接。一是以私营企业、个体工商户和事业单位为重点，抓好基本养老保险和失业保险扩面工作，基本养老保险和失业保险参保人数均达到20万人；强化社保费征缴，确保离退休人员基本养老金按时足额发放、符合条件的失业人员享受失业保险待遇。二是规范城市低保制度，切实做到应保尽保。加强对享受低保人员的动态管理，按照省政府要求，适当提高补助标准。三是推进医疗保险制度改革，基本医疗保险参保人数达到25万人，医保网络要全部启动、联网。四是继续加强社区居委会基础设施建设，确保社区办公室和“一站三室”全面达到省定标准，为社区更好地发挥在社会保障中的作用创造条件。

　　切实关心人民群众生活，广泛深入地开展“送温暖、献爱心”扶贫帮困活动。街道、社区居委会要对城市重点贫困户进一步普查，将城市低保户中的特困户和生活困难的下岗职工作为重点帮扶对象，重点对特困学生、大病无钱就医家庭进行救助。继续采取包乡带村的办法，搞好农村扶贫帮困，为农村特困户建房150套，在阜、彰两县各建一所区域性中心敬老院。继续实施扶贫开发工程，抓好60个村整村开发式扶贫和440户农民移民扶贫。

　　（十）加强精神文明建设，全面发展各项社会事业

　　大力弘扬抗击非典精神和载人航天精神，为转型复兴提供强大的精神动力。以创建文明城市、文明行业、文明单位为载体，坚持贴近实际、贴近生活、贴近群众，立足于办实事、求实效，广泛开展群众性精神文明创建活动，不断提高全市人民的思想道德素质和城乡文明程度，创造良好的社会环境。

　　以建设具有地域特色文化为目标，抓好“玉龙”文化月和首届职工艺术节等群众性文化活动，积极备战省第六届文化艺术节。推进有线电视“村村通”工程，实现全地区有线电视联网，探索实施广播电视数字化工程。广泛开展以篮球项目为龙头的全民健身活动，抓好篮球、赛艇、举重三个国家后备人才基地建设。继续搞好篮球城建设，发展篮球产业。以篮球为媒介，推进对外合资合作。建立健全疾病信息网络体系、疾病预防控制体系和医疗救治体系，提高公共卫生服务水平和突发性公共卫生事件应急能力。社区卫生服务覆盖率达95%以上。加强农村公共卫生体系建设，争取阜蒙县被国家批准为新型农村合作医疗试点县。大力开展爱国卫生运动，创建省级卫生城市。强化人口与计划生育综合治理工作，提高出生人口素质。加强国防教育，继续做好双拥工作，进一步落实优抚和安置政策，巩固和发展军政军民团结。关心和支持老龄、残疾人事业，做好档案管理、续修市志、地震预测预报等工作。

　　（十一）加强民主和法制建设，切实维护社会稳定

　　自觉接受人民代表大会及其常委会的依法监督和人民政协的民主监督，认真办理人大代表议案、建议和政协提案。加强与民主党派、工商联、无党派人士和人民团体的联系，重视并听取其意见和建议。支持群团组织工作，充分发挥工会、共青团、妇联和科协等组织在安置就业、扶贫和科技进步等方面的积极作用。推进“四五”普法教育，提高全民法律素质。加强依法行政，建立健全行政执法责任制和执法过错追究制，做到严格执法、公正执法、文明执法。认真做好行政复议应诉和仲裁工作。继续整顿和规范市场经济秩序，深入开展“打假”斗争，严厉打击逃税、逃债行为，建设诚信社会。全面贯彻党的民族政策，加强民族团结，积极支持和帮助少数民族地区经济加快发展。依法加强对宗教事务的管理。继续做好外事、侨务、对台、人防、民防工作。

　　坚持打防结合、预防为主，全面落实社会治安综合治理的各项措施，确保全市的政治稳定和社会稳定。深入开展“严打”整治斗争，依法严厉打击各种刑事犯罪和经济犯罪活动，采取有力措施，及时处理和解决危害社会公共安全的犯罪问题，同“法轮功”等邪教组织作坚决斗争。认真做好国家安全工作。高度重视人民群众来信来访，完善领导包案责任制，妥善办理涉及群众利益的群体访事件，努力营造和谐稳定的社会环境。严格落实防火和安全生产责任制，加大消防和安全生产监督执法力度，坚决遏制重特大事故的发生。

　　（十二）转变作风，提高效率，进一步加强政府自身建设

　　新的形势和任务，对各级政府以及公务员队伍提出了更新、更高的要求。为此，必须加强自身建设，更好地肩负起阜新转型复兴的神圣使命。第一，解放思想，始终保持奋发向上的精神状态。各级政府及其工作人员都要着眼于跨越式发展，充分把握和利用好难得的三大历史性机遇，树立路在脚下、事在人为的精神，坚决克服安于现状、小进即满、不求进取的思想，创造性开展工作，在推进转型复兴中不断提高领导经济工作能力和驾驭市场经济能力。第二，以人为本，密切同人民群众的血肉联系。各级政府要把执政为民作为各项工作的着眼点和归宿，制定为民谋利的具体计划，每年多办几件关系群众切身利益的好事实事。各级领导干部要体察民情，多到条件差、困难多、群众意见大的地方去，在解决热点、难点问题中不断提高应对复杂局面的能力。第三，开展“环境建设年”活动，提高政府工作效率和服务水平。结合省委、省政府开展的环境年建设活动，在全市开展以减少审批环节、提高工作效率、提高服务水平为重点的环境建设年活动。进一步做实“一站式”办公、“保姆式”服务，着力营造一流的经济发展软环境。对于给全市软环境建设造成不良影响的单位和个人，予以严肃查处。以贯彻《行政许可法》为契机，继续深化行政审批制度改革，建立市行政服务中心，切实减少审批环节，简化审批程序，依法规范政府行为。畅通政务公开渠道，重点抓好建设工程招投标、经营性国有土地使用权挂牌招标、政府采购等重大事项的公开，主动接受社会各界的监督。第四，强化责任，推进各项工作的落实。对今年政府各项工作任务，要按照科学分解、重点突出的原则，将任务指标迅速逐级分解到位。对事关转型复兴全局的重点、难点工作，各级政府、各部门的一把手要逐个问题、逐个阶段、逐个部位地抓好落实，达到“干一件，成一件，件件落实”的效果。强化责任目标完成情况的科学考评，对于不干、干不好的问题要进行责任追究。第五，廉洁自律，树立公正廉洁的政府形象。各级政府工作人员要牢记“两个务必”，厉行节约，勤俭办事。全面落实党风廉政建设责任制，一级抓一级，层层抓落实。认真做好纠正部门和行业不正之风工作。对以权谋私、贪污受贿的腐败分子，坚决予以查处。要通过政府自身建设，以过硬的自身素质，真正在人民群众中树立起政府机关的良好形象。

　　各位代表，加快推进阜新转型复兴，使命光荣，任重道远。让我们高举邓小平理论和“三个代表”重要思想的伟大旗帜，紧密团结在以胡锦涛同志为总书记的党中央周围，在市委的正确领导下，凝聚全市人民的力量和智慧，万众一心，抢抓机遇，发愤图强，开拓创新，向着阜新转型复兴的宏伟目标奋勇前进！
